# Predicting anticipated benefit from an extended consultation to personalise care in multimorbidity: a development and internal validation study of a prioritisation algorithm in general practice

**DOI:** 10.3399/BJGP.2023.0114

**Published:** 2024-04-16

**Authors:** Mieke JL Bogerd, Collin JC Exmann, Pauline Slottje, Jettie Bont, Hein PJ Van Hout

**Affiliations:** Department of General Practice, Amsterdam UMC, Vrije Universiteit Amsterdam, and Amsterdam Public Health Research Institute, Amsterdam, the Netherlands.; Department of General Practice, Amsterdam UMC, Vrije Universiteit Amsterdam, and Amsterdam Public Health Research Institute, Amsterdam, the Netherlands.; Department of General Practice, Amsterdam UMC, Vrije Universiteit Amsterdam, and Amsterdam Public Health Research Institute, Amsterdam, the Netherlands.; Department of General Practice, Amsterdam UMC, Vrije Universiteit Amsterdam, and Amsterdam Public Health Research Institute, Amsterdam, the Netherlands; Department of General Practice, Amsterdam UMC, Vrije Universiteit Amsterdam, and Amsterdam Public Health Research Institute, Amsterdam, the Netherlands.

**Keywords:** general practice, multimorbidity, person-centred care, primary care

## Abstract

**Background:**

Persons with multimorbidity may gain from person-centred care compared with the current protocolised chronic-disease management in Dutch general practice. Given time constraints and limited resources, it is essential to prioritise those most in need of an assessment of person-centred chronic-care needs.

**Aim:**

To develop and validate a prioritisation algorithm based on routine electronic medical record (EMR) data that distinguishes between patients with multimorbidity who would, and those who would not, benefit from an extended person-centred consultation to assess person-centred chronic-care needs, as judged by GPs.

**Design and setting:**

A mixed-methods study was conducted in five general practices in the north-west region of the Netherlands. Four out of the five practices were situated in rural areas.

**Method:**

Multivariable logistic regression using EMR data to predict the GPs’ judgement on patients’ anticipated benefit from an extended consultation, as well as a thematic analysis of a focus group exploring GPs’ clinical reasoning for this judgement were conducted. Internal validation was performed using 10-fold cross-validation. Multimorbidity was defined as the presence of ≥3 chronic conditions.

**Results:**

In total, EMRs from 1032 patients were included in the analysis; of these, 352 (34.1%) were judged to have anticipated benefit. The model’s cross-validated C-statistic was 0.72 (95% confidence interval = 0.70 to 0.75). Calibration was good. Presence of home visit(s) and history of myocardial infarction were associated with anticipated benefit. Thematic analysis revealed three dimensions feeding anticipated benefit: GPs’ cause for concern, patients’ mindset regarding their conditions, and balance between received care/expected care needed.

**Conclusion:**

This algorithm may facilitate automated prioritisation, potentially avoiding the need for GPs to personally triage the whole practice population that has multimorbidity. However, external validation of the algorithm and evaluation of actual benefit of consultation is recommended before implementation.

## Introduction

Persons with multiple chronic conditions, also called multimorbidity, often need more frequent general practice consultations and require more complex care than those with a single condition.^1^^,^^2^ Their care frequently involves multiple care professionals, making it harder to achieve a comprehensive overview and coordinated management,^3^^,^^4^ and they account for a growing proportion of the general practice workload.^5^ Despite the increasing number of people with multimorbidity, the delivery of chronic care in general practice is usually organised around single diseases.^6^

In the Netherlands, disease-management programmes (DMPs) were developed for diabetes mellitus, asthma and chronic obstructive pulmonary disease, and cardiovascular diseases. These disease-specific programmes have been shown to improve lifestyle (for example, physical activity) and short-term health indicators (for example, blood–sugar levels).^7^ However, in the case of multimorbidity, treating each patient’s chronic disease separately may lead to adverse outcomes and will increase consultations with general practice.^8^ Also, as DMPs include scheduled standard check-up appointments, there is little room for tailored care agreements. A shift towards a more person-centred care approach that transcends individual diseases is advocated.^2^

Although the rationale for person-centred chronic care has been clearly defined,^6^ evidence for potential benefits and risks of such care approaches for persons with multimorbidity is mixed. A Cochrane systematic review of person-centred initiatives reported improved mental health outcomes and improved healthcare provider behaviour, but showed an inconclusive effect on participants’ physical health.^9^ A pragmatic cluster randomised trial did not find effects on health-related quality of life;^10^ however, the process evaluation of the trial showed certain implementation failures and the authors suggested potential intervention improvements, including regarding the selection of patients.^11^^,^^12^

**Table:** How this fits in

Current disease management programmes usually focus on one illness and are, therefore, less suited to persons with multimorbidity, for whom more person-centred, comprehensive chronic care is recommended. Given the time constraints and limited resources in general practice, it is essential to prioritise those most in need of an assessment of person-centred chronic-care needs. This study presents an algorithm that uses electronic medical record data predicting anticipated benefit, as judged by GPs, which performs reasonably well in prioritising patients with multimorbidity. This algorithm may help GPs to target comprehensive care efforts by facilitating automated prioritisation, potentially avoiding the need for them to personally triage the whole practice population.

It has been proposed that consultation lengths for patients with multiple chronic conditions should be extended.^13^^,^^14^ The GP and patient would then have time to discuss all chronic conditions and related problems in context, and to make tailored care agreements. In a Dutch participatory action research project that aimed to develop a person-centred chronic-care approach for persons with multimorbidity,^15^ GPs introduced the idea of an extended person-centred consultation (EPCC). This consultation can be applied to certain patients as a starting point for organising person-centred, tailored care. However, as persons with multimorbidity constitute a diverse spectrum, it remains unclear which specific individuals can be effectively targeted.

Given the time constraints and limited resources in general practice, it is essential to prioritise those most in need of an EPCC to assess person-centred chronic-care needs and organise care. In addition, GPs could save time if there was a way to prioritise these individuals using the information already available in medical records. For instance, previous studies found that contact frequency with the general practice,^16^^–^18 the number of chronic conditions,^19^ and the presence of certain specific chronic conditions^20^ may be relevant. To help GPs prioritise patients, this study aimed to develop and internally validate a prioritisation algorithm using routine medical record data that distinguishes between those patients with multimorbidity who would, and would not, benefit from an EPCC, as judged by GPs.

## Method

### Setting

This study was part of COPILOT, a Dutch participatory action research project, initiated by GPs^15^ who introduced the idea of an EPCC. Instead of the regular 10-minute consultation, the EPCC may last up to 30 minutes so GPs and patients can discuss in depth the patient’s health status, healthcare needs (from the perspectives of both the patient and the GP), and person-centred goals.

The current study was conducted in March and April 2021 in five general practices situated in the north-west region of the Netherlands. Four out of the five practices were situated in rural areas. In the Netherlands, all community-dwelling citizens are registered with one general practice and the GP functions as a gatekeeper for specialised care. Management and follow-up within DMPs are based on the practice guidelines of the Dutch College of General Practitioners. In these programmes, patient care is mainly provided by practice nurses, supervised by GPs.

### Design

In this cross-sectional study, mixed methods were used, comprising:

- diagnostic prediction modelling to predict GPs’ judgement of who would, and would not, benefit from an EPCC using electronic medical record (EMR) data; and
- thematic analysis of a focus group exploring GPs’ clinical reasoning for this judgement.

The Transparent Reporting of a multivariable prediction model for Individual Prognosis Or Diagnosis (TRIPOD)^21^ process was followed.

### Participants: GPs

Five GPs from five different practices participated in the study reported here. The five GPs were conveniently sampled. All were partner GPs with 13–20 years’ work experience and had been working at these practices for 10–19 years. Practice types included solo, duo, and group practices. One practice (practice 3) typically served patients with low socioeconomic status. In the other four practices, the socioeconomic status of patients was more diverse. All participating GPs were closely involved from the start of the COPILOT project and, as such, had extensive experience with the EPCC.

### Procedure

Patients were eligible if they were adults deemed mentally competent and had multimorbidity, which was defined as three or more chronic conditions (Supplementary Table S1). Dutch GPs register diagnoses using the International Classification of Primary Care.^22^ Patients were excluded if they were:

- terminally ill;
- diagnosed with dementia (P70) or mental retardation (P85);
- severely hearing or visually impaired (H86 and F94 respectively); or
- enrolled in a care programme for vulnerable older persons.

A uniform search query in the EMR was developed for GPs to select potentially eligible patients. GPs were allowed to exclude selected persons and checked whether selected persons met the inclusion and exclusion criteria, based on their personal knowledge. They then used a traffic-light system (low — green, moderate — amber, and high — red) to score the anticipated benefit of these patients having an EPCC. This system is well known to GPs and is frequently used in triage.^23^

Pseudonymised routine care data from the EMR covering the years 2019 and 2020 were used. Initial analyses indicated a better predictive performance of the 2-year lookback period over a 1-year period (data not shown). Patients were able to opt out, in which case their data from the EMR were not shared with the researchers or used in the analyses. Comprehensive information regarding the study and the opt-out protocol concerning their EMR data was disseminated to patients through an invitation letter from their GP and the study’s information leaflet. Included EMR systems were Medicom (four practices) and ZorgDossier (one practice). GPs’ scores were collected in March and April 2021.

### Candidate predictors for anticipated benefit, as judged by GPs

Demographic variables (for example, gender and age) and information on the patients’ medical history relating to their chronic diagnoses (Supplementary Table S1) were included. Variables regarding the absence or presence of each chronic diagnosis, along with a count variable of all chronic diagnoses, were included. The number of different chronically prescribed medications (defined at the fifth anatomical therapeutic chemical [ATC] level) was included; such medications were defined as those that were:^24^

- prescribed at least three times per year (with the last prescription being in the second half of the year);
- labelled as ‘chronic’; or
- registered as ‘repeat >0’.

Regarding the frequencies of contact with general practice, the number of the following that were registered in the EMR were included:

- home visits;
- face-to-face consultations;
- telephone calls;
- contacts registered for repeat prescriptions; and
- emails.

A maximum of one contact for each contact type per day was counted. The number of other contacts (for example, administrative actions, providing files to third parties) was also counted.

### Outcome definition

The primary outcome was the GP’s clinical judgement of the presence or absence of an anticipated benefit of an EPCC for the patient. Initially, GPs were asked to score their patients using the traffic-light system, but the outcome measure was dichotomised given the research objective of distinguishing between patients whom GPs anticipated would, and would not, benefit from an EPCC. The ‘moderate’ and ‘high’ categories of anticipated benefit were merged; this resulted in an increase in the number of patients per outcome category, which favoured the number of predictors that could be included in the analyses. Patients whose score was ‘low’ were deemed to have no anticipated benefit when scores were dichotomised.

### Sample size

Participating GPs expected to score approximately 40% of their patients with multimorbidity as having an anticipated benefit. Using the multivariate analyses rule of thumb that one candidate predictor can be included for every 10 cases in the smallest group of the outcome variable, in a sample of 1000 participants with 40% (*n* = 400) anticipated benefit, 40 candidate variables could be studied simultaneously. The authors deemed this appropriate.

### Quantitative analysis

Logistic regression^25^ was used to develop the prediction model. Data were analysed using IBM SPSS software (version 26). Backward stepwise deletion was applied with a threshold of *P*<0.1.^26^ The high number of potential predictors meant that a pre-selection to the backward deletion procedure was performed, selecting candidate predictors with univariate associations of *P*<0.2 into a multivariate model.^27^

Prior to these analyses, outliers and regression assumptions including multicollinearity, normality, and log linearity were checked.^27^ In the case of log linearity, candidate predictors were converted to quartiles, tertiles, or were dichotomised based on their interquartile ranges. Discriminatory performance of the model was evaluated using the C-statistic. Calibration was assessed by visual inspection of the calibration plot and by considering the intercept and slope. The results were internally validated using a 10-fold cross-validation procedure.^21^ This procedure implied the random assignment of participants to one of 10 groups and running the whole analysis 10 times, with the exclusion of one group each time, while evaluating the developed model in this excluded group. This procedure contained all steps taken in the analysis so, in every fold, the variable selection based on univariate association and backward deletion was repeated.

For this prediction model to be useful in practice as a first step in a triage process, it should distinguish accurately between patients who would, and would not, benefit from an EPCC, as judged by GPs. To determine the optimal cut-off value, therefore, the diagnostic accuracy measures were considered for multiple cut-off values in the following order:

- negative predictive values (NPVs);
- number of patients predicted by the model as having no anticipated benefit when GPs judged them as having high anticipated benefit; and
- the Youden index.

The Youden index (sensitivity + specificity − 1) optimises the model’s differentiating ability when equal weight is given to sensitivity and specificity.^28^ Other accuracy measures, including sensitivity, specificity, and positive predictive value, were also calculated. In addition, a decision curve analysis was performed, which calculates a clinical net benefit for prediction models or diagnostic tests in comparison with default strategies of treating all or no patients.^29^

Finally, sensitivity analyses were performed to explore possible inter-practice and inter-GP variation in judging anticipated benefit, by alternately excluding one practice.

### Qualitative analysis

A semi-structured focus group was conducted to explore GPs’ thoughts concerning their clinical judgements of patients’ anticipated benefit from an EPCC. As the concept of anticipated benefit for a person-centred consultation is new and was not clearly defined, these findings complemented the quantitative section by exploring the reasons why GPs prioritised certain patients with multimorbidity for the EPCC, but not others. The focus group was audio-taped and transcribed verbatim, and comments were pseudonymised; two researchers conducted independent analyses using data analysis software MaxQDA (2020). Data analysis followed the coding template of the Gioia methodology with an open coding procedure;^30^ this method was chosen because of its usefulness to develop a data structure of higher-order and lower-order concepts, which facilitated a better understanding of the quantitative results.

## Results

### Study population and outcome

**Figure 1.**
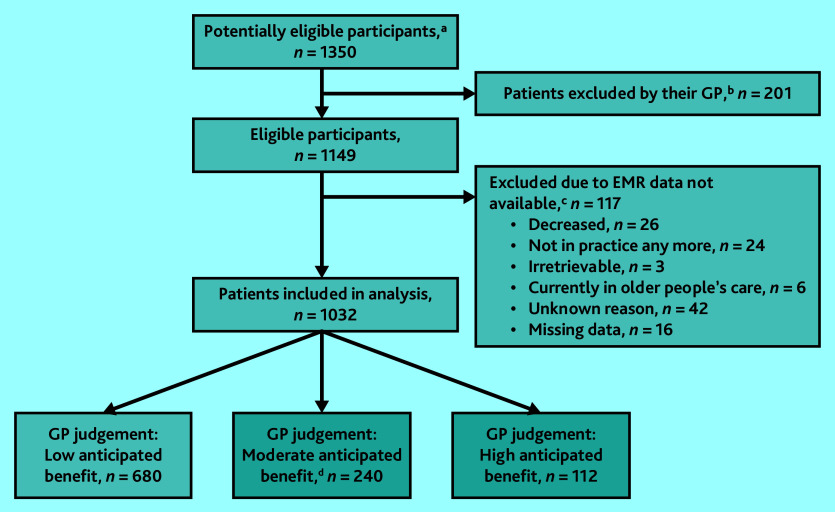
Flowchart sample selection. ^a^Potentially eligible participants were selected based on an EMR search query that included data from the years 2017 and 2018 (baseline measurement [T0] of the COPILOT project). ^b^At T0 of the COPILOT project, GPs checked whether these potentially eligible patients met the inclusion or exclusion criteria based on their personal knowledge. This was done as EMR data relating to the criteria might not be accurate. If necessary, GPs were allowed to exclude patients on this basis. ^c^This study utilised data from the COPILOT project, an action-based research study aimed at co-creating and piloting a proactive, person-centred, chronic-care approach for patients with multimorbidity. Data were collected during three measurement waves: 2019 (T0), 2020 (T1), and 2021 (T2). For the current study, T2 data were used. However, some patients were lost to follow-up because of reasons specified in this figure or excluded because of the unavailability of EMR data. ^d^Patients included in the shaded boxes (moderate and high anticipated benefit) were merged in the analysis. EMR = electronic medical record.

No patients opted out of the study and EMR data for 1032 patients were included in the analysis. The sample selection flowchart is presented in Figure 1. Overall, 352 (34.1%) patients were scored by their GP as having moderate (*n* = 240) or high (*n* = 112) anticipated benefit from an EPCC. Descriptive statistics of the patient sample are shown in Table 1, stratified by the dichotomous outcome.

**Table 1.** Characteristics of patients included in analysis, by anticipated benefit category

**Characteristic**	**Whole sample**	**Anticipated benefit**	**No anticipated benefit**
Total	1032	352	680

Demographic			
Median age, years (IQR)	70 (62–77)	71 (63–77)	69 (62–77)
Men, *n* (%)	477 (46.2)	183 (52.0)	294 (43.2)
Median number of chronic conditions, *n* (IQR)	7 (5–9)	7 (5–9)	6 (5–8)
Median number of different chronically prescribed medications, *n* (IQR)^a^	5 (3–7)	6 (4–8)	4 (2–7)

Median number of contacts with general practice over 2 years, *n* (IQR)			
Home visits	0 (0–0)	0 (0–1)	0 (0–0)
Face-to-face consultations	12 (7–18)	14 (9–21)	11 (6–17)
Telephone calls	6 (3–10)	6 (4–12)	5 (3–9)
Repeat prescriptions^b^	0 (0–3)	2 (0–11)	0 (0–1)
Short consultations	0 (0–1)	0 (0–1)	0 (0–1)
Other contacts	15 (10–22)	16 (10–24)	14 (9–21)
Emails	0 (0–0)	0 (0–0)	0 (0–0)

Clusters of chronic diagnoses, *n* (%)^c^			
Cancer	356 (34.5)	138 (39.2)	218 (32.1)
Diabetes	298 (28.9)	121 (34.4)	177 (26.0)
Urinary	315 (30.5)	105 (29.8)	210 (30.9)
HIV/AIDS	<5	<5	<5
Adiposity	71 (6.9)	24 (6.8)	47 (6.9)
Eye disease	62 (6.0)	22 (6.3)	40 (5.9)
Psoriasis	99 (9.6)	26 (7.4)	73 (10.7)
Tobacco abuse	371 (35.9)	103 (29.3)	268 (39.4)
Alcohol abuse	72 (7.0)	25 (7.1)	47 (6.9)
Bowel disorders	150 (14.5)	51 (14.5)	99 (14.6)
Cardiovascular	909 (88.1)	328 (93.2)	581 (85.4)
Musculoskeletal	747 (72.4)	245 (69.6)	502 (73.8)
Neurologic	329 (31.9)	105 (29.8)	224 (33.0)
Psychological	499 (48.4)	171 (48.6)	328 (48.2)
Respiratory	286 (27.7)	97 (27.6)	189 (27.8)
Thyroid	157 (15.2)	57 (16.2)	100 (14.7)

a *Number of medications (defined at fifth anatomical therapeutic chemical level) chronically prescribed in 2020. Chronically prescribed medications were defined as those that were: prescribed a minimum of three times per year (of which one was prescribed in the second half of the year), labelled as ‘chronic’, or registered as ‘repeat >0’.*

b *Number of contacts registered for repeated prescriptions in 2019 and 2020.*

c *See Supplementary Table S1 for all included ICPC codes per cluster. AIDS = acquired immune deficiency syndrome. HIV = human immunodeficiency virus. ICPC = International Classification of Primary Care. IQR = interquartile range.*

### Prediction model development and internal validation

Based on univariate regression models, 24 chronic diagnoses were included in the preliminary prediction model. The full multivariate model is shown in Supplementary Table S2. After backward selection, the final multivariate prediction model resulted in an apparent C-statistic of 0.777 (Table 2). The receiver operating curve is presented in Supplementary Figure S1. The model also retained its predictive power after cross-validation, with a cross-validated C-statistic of 0.724. Calibration was good (Supplementary Figure S2). Diagnostic accuracy measures for the model at different cut-off values are presented in Table 3. Presence of home visit(s) in the previous 2 years was the strongest predictor; myocardial infarction and cerebrovascular accidents were also found to be significant in this model (Table 2).

**Table 2.** Model predicting anticipated consultation benefit (*n* = 1032)

	**Final model**			

	**B**	**Sig**	**Odds**	**95% CI**
Gender	0.363	0.019	1.44	1.06 to 1.95

Number of different chronically used medications^a^				
0–3	−0.619	0.006	0.54	0.35 to 0.84
4–5	−0.317	0.152	0.73	0.47 to 1.12
6–7	−0.169	0.450	0.84	0.55 to 1.31

Contact with general practice over 2 years				
Consultations	0.015	0.060	1.01	1.00 to 1.03
Repeat prescriptions^b^				
0	−1.833	0.000	0.16	0.11 to 0.23
1–3	−1.970	0.000	0.14	0.09 to 0.22
Home visits, dichotomised	0.735	0.000	2.09	1.45 to 3.00

Diagnoses^c^				
Cancer	0.302	0.053	1.35	1.00 to 1.84
Infectious heart disease	1.470	0.029	4.35	1.16 to 16.28
Myocardial infarction	0.735	0.002	2.09	1.31 to 3.33
Cerebrovascular accident	0.390	0.074	1.48	0.96 to 2.26
Cox arthrosis	−0.510	0.021	0.60	0.39 to 0.93
Psoriasis	−0.565	0.037	0.57	0.33 to 0.97

Z episode^d^	−0.406	0.020	0.67	0.47 to 0.94

Constant	0.420	0.144	1.52	0.00 to 0.00

Model summary	Apparent value	Cross-validated value		95% CI
C-statistic	0.777	0.724		0.700 to 0.747

a *Reference category was ≥8 medications.*

b *Reference category was ≥4 repeated drug prescriptions.*

c *See Supplementary Table S1 for all included ICPC codes per cluster.*

d *Presence of ICPC code(s) from the Z chapter (social problems, see Supplementary Table S3). B = beta. ICPC = International Classification of Primary Care. Sig = significance, the* P*-value.*

**Table 3.** Diagnostic accuracy measures of the developed model (*n* = 1032) for predicting anticipated benefit from an EPCC at different cut-off values

Cut-off value	0.1	0.2	0.3^a^	0.4^a^	0.5	0.6	0.7	0.8	0.9
NPV, %	96.3	87.7	83.1	79.7	77.2	73.1	68.7	67.3	66.2
False negative in the high anticipated benefit score group, %	0.0	7.1	17.9	25.9	33.9	54.5	75.0	87.5	97.3
Youden index	0.07	0.35	0.43	0.41	0.38	0.27	0.11	0.06	0.02
Patients predicted by the model as having anticipated benefit (moderate and high), % of total	94.8	64.4	44.4	32.7	24.4	16.8	8.1	4.3	0.7
Patients scored by the GP as having anticipated benefit (moderate and high), % of total	34.1	34.1	34.1	34.1	34.1	34.1	34.1	34.1	34.1
Sensitivity, %	99.4	87.2	72.4	59.9	49.9	34.4	15.6	8.2	1.7
Specificity, %	7.6	47.4	70.1	81.5	88.5	92.4	95.7	97.8	99.9
PPV, %	35.8	46.2	55.7	62.6	69.0	69.9	65.5	65.9	85.7

a *The cut-off values 0.3 and 0.4 were considered, with the cut-off value 0.3 chosen as optimal.*

*EPCC = extended person-centred consultation. NPV = negative predictive value. PPV = positive predictive value.*

### Optimal cut-off value

**Figure 2.**
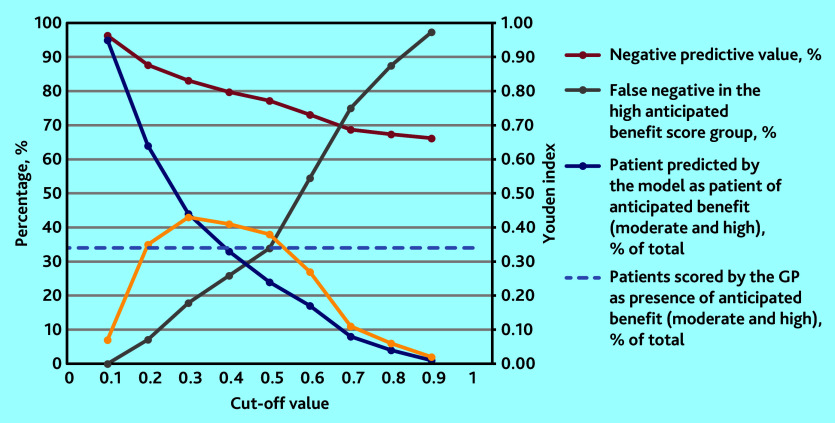
Measures to determine the optimal cut-off value of the diagnostic model for anticipated benefit from an EPCC. EPCC = extended person-centred consultation. NPV = negative predictive value.

In Table 3, all diagnostic accuracy measures for every cut-off value are reported. In Figure 2, only the measures used to determine the optimal cut-off value are shown; the cut-off values 0.3 and 0.4 were considered and the cut-off value 0.3 was chosen as optimal. Compared with the cut-off value of 0.4, the false negative percentage in the high anticipated benefit group was much lower at the cut-off value 0.3 (18% versus 26%); the NPV and Youden index remained roughly the same for the two cut-off points. In addition, the decision curve analysis graphically shows that the algorithm can add net benefit to the prediction of anticipated benefit, with the threshold probability ranging from 9% to 100% (Supplementary Figure S3).

### Sensitivity analysis

Sensitivity analyses (Supplementary Table S4) showed no major differences in diagnostic accuracy measures when alternately excluding practices one to four. When excluding practice five, these measures did differ substantially. All diagnostic accuracy measures differed (Table S4).

### Focus group

Four out of five GPs participated in the focus group. One of the five GPs sadly passed away during the study. Coding resulted in three aggregate dimensions that were of importance for the concept of anticipated benefit:

- GP’s causes for concern,
- patient’s mindset regarding conditions, and
- the balance between received care and expected care needed in general practice.

The data structure can be found in Supplementary Figure S4.

Causes for concern — which could be based on complexity, instability, or the GP’s gut feeling — meant that patients considered to fall into this category had a higher chance of benefiting from the consultation; it described their situation, which was often quite frail. In a description of one such case, a GP stated:

*‘He has multiple conditions, can hardly see, and has multiple problems, which could potentially make him vulnerable, although he currently manages it all quite well … But the balance is fragile.’*

(GP4)

The patient’s mindset also mattered according to the GPs. This considered how people saw health as part of their life or how they handled it — for example, being too modest to call for the GP’s attention, or calling them for the smallest complaint. Patients who were considered to be overly modest were identified for anticipated benefit:

*‘People who think “The doctor is busy enough, I’m not going to bother him”, those are the ones really at risk!’*

(GP4)

Finally, the balance between received care and expected care needs in general practice was a dimension of interest. GPs noted that DMPs could lead to new diagnoses as they could provide an extra moment for patients to talk about other complaints they were experiencing, which could lead to extensive medical histories being noted. Conversely, however, some patients who did not regularly visit the GP could benefit; these patients might lack the ability to organise their care and ask for help in a timely fashion:

*‘They may come faithfully for their check-ups, but they don’t come in with other complaints.’*

(GP2)

## Discussion

### Summary

This study is embedded in a participatory action research project and responded to a need expressed by GPs for a prioritisation tool — preferably based on readily available routine EMR data — they could use to decide who to invite first for an extended consultation to assess person-centred chronic-care needs and organise care. The developed algorithm to distinguish between patients with multimorbidity who would, and would not, benefit from such a consultation, as judged by GPs, performed satisfactorily and retained its discriminative power after internal validation.

The focus group analysis revealed three dimensions that fed anticipated benefit as judged by GPs: GPs’ cause for concern, the patients’ mindset regarding their conditions, and the balance between received care and expected care needs in general practice.

### Strengths and limitations

One major strength was the use of mixed methods to develop the model (based on EMR data) and interpret the concept of anticipated benefit (based on a focus group). Also, the use of routine EMR data promoted the applicability of the algorithm when integrated into a tool in practice.

One limitation regarding the generalisability of the findings was that only five GPs from different practices in one region participated in the scoring process, with only four attending the focus group session. Although sensitivity analyses, which involved alternately excluding one of the practices, did not show large differences in diagnostic accuracy measures for the most part, this was evident when one particular practice was excluded. Potential explanations for this inter-practice difference in model performance include sample size, case mix of patients, the GP’s information system (EMR), and registration behaviour, as well as the subjective clinical judgement of the GP. External validity could be improved by including GPs from diverse backgrounds and practices, who are also familiar with the concept of an EPCC and anticipated benefit. In addition, interrater reliability could have been investigated by having multiple GPs in the same practice score the same patients for anticipated benefit.

The relatively small sample size could impact the performance of the algorithm when applied to diverse populations or different settings; this highlights the significance of external validation before implementation. In addition, EMR data seemed to be unable to capture the dimension relating to a person’s mindset regarding their conditions and the patients’ perspective on anticipated benefit was not taken into account.

The dichotomisation of the outcome measure could also have resulted in the potential loss of information; however, the decision to merge the moderate and high anticipated-benefit categories allowed for a clearer distinction between patients who were, and were not, anticipated to benefit from EPCCs. It also remains to be seen whether any anticipated benefit of an EPCC would lead to real benefit.

### Comparison with existing literature

To the authors’ knowledge, this is the first study to predict the clinical judgement of GPs with regard to who to prioritise for an extended consultation in patients with multimorbidity as a starting point for organising person-centred chronic care. One of the dimensions that emerged from the thematic analysis — that is, balance between received care and expected care needs from general practice — might have been captured with the information concerning the frequencies of contact with general practice. Prior studies showed that frequent attendance in general practice was associated with higher care needs^31^^,^^32^ and, as such, may correspond with higher consultation benefit. However, participating GPs also pointed to infrequent attenders, who might lack the ability to organise their care and ask for help in a timely manner. Concerning the general population, published studies have showed that inviting infrequent attenders is often not an effective way of providing care,^33^ and infrequent attenders are often healthier and self-reliant.^34^

Besides frequency, the type of the contact was informative: the presence of one or more home visits in the previous 2 years was the strongest predictor. This makes sense as it concerns individuals who are unable to come to the practice.

Another dimension revealed through thematic analysis was the GPs’ causes for concern, which was captured by specific diseases, the number of chronically prescribed medications, and age. This dimension is consistent with literature that has suggested complexity and social functioning as a reason for person-centred approaches.^3^ Specific diseases, such as cerebrovascular accident, myocardial infarction, and cancer can, arguably, be associated with anticipated benefit. Others, such as psoriasis and migraine, were negatively associated with anticipated benefit; from the GP’s perspective, these conditions may not necessarily be a focus for EPCCs as patients with these often manage these illnesses themselves or are treated by medical specialists.

### Implications for research and practice

This algorithm intended to inform GPs on the likelihood of a patient’s anticipated benefit from an EPCC to assess person-centred chronic-care needs and organise care. The algorithm may facilitate automated prioritisation, potentially avoiding the need for GPs to personally triage the whole practice population with multimorbidity, and support GPs in efficiently determining whom to prioritise for an EPCC. Optimally, the tool should be utilised both as a panel (that is, the GP receives an overview of all their patients, alongside their predicted likelihood of benefit, aiding the GP in inviting patients for consultations) and at an individual level, for example, by incorporating a pop-up feature in the EMR system. However, before application, external validation of the algorithm is recommended. Moreover, future research should compare the anticipated benefit (before) and perceived benefit (after) of such an extended consultation, and evaluate the effects on subsequent healthcare utilisation, both within and outside of general practice.

## Data

For privacy reasons, the data cannot be made publicly available. The authors do not have ethical permission or patient consent to share the full data. The dataset analysed for the current study is available from the corresponding author upon reasonable request.
